# The effect of smoking on tumor immunoediting: Friend or foe?

**DOI:** 10.18332/tid/189302

**Published:** 2024-06-17

**Authors:** Yixia Jiang, Hequan Li

**Affiliations:** 1 Department of Respiratory Diseases, The First Affiliated Hospital of Zhejiang University School of Medicine, Hangzhou, China

**Keywords:** smoking, tumor immunoediting, immune surveillance, neoantigen, immunotherapy

## Abstract

The recognition of smoking as an independent risk factor for lung cancer has become a widely accepted within the realm of respiratory medicine. The emergence of tumor immunotherapy has notably enhanced the prognosis for numerous late-stage cancer patients. Nevertheless, some studies have noted a tendency for lung cancer patients who smoke to derive greater benefit from immunotherapy. This observation has sparked increased interest in the interaction between smoking and the immune response to tumors in lung cancer. The concept of cancer immunoediting has shed light on the intricate and nuanced relationship between the immune system and tumors. Starting from the perspectives of immune surveillance, immune equilibrium, and immune evasion, this narrative review explores how smoking undermines the immune response against tumor cells and induces the generation of tumor neoantigens, and examines other behaviors that trigger tumor immune evasion. By elucidating these aspects, the review concludes that smoking is not conducive to tumor immunoediting.

## INTRODUCTION

It is widely recognized that smoking has a detrimental impact on human health^1^. Despite increasing efforts to regulate tobacco sales and curb smoking habits, it accounted for 71000 deaths worldwide and contributed to 7.3% of disability-adjusted life years in 2017^2^. Epidemiological studies have consistently linked smoking to elevated rates of lung cancer incidence and mortality, with evidence suggesting that quitting smoking can confer benefits even after a lung cancer diagnosis^3,4^. However, exactly how smoking works in cancer development remains inconclusive. With the advent of tumor immunotherapy, researchers have turned more attention to the relationship between smoking and tumor immunity. Recent findings indicate that non-small cell lung cancer patients with a history of smoking may exhibit an improved response to anti-PD-L1 therapy compared to non-smoking patients, suggesting a potential impact of smoking on tumor prognosis^5^. This raises questions about smoking’s role as a tumor risk factor. Immunity and tumors have always been inseparable topics. The concept of immune surveillance has evolved into the immunoediting theory, which posits a dynamic interaction between tumors and the immune system^6^. Immunoediting theory believes that the immune system is a double-edged sword on tumors^7^. In this theory, the relationship between tumors and immune systems can be divided into three stages^8^. In the early stage of tumorigenesis, the immune system mainly manifests as elimination. However, a subset of tumor cells has the innate or acquired ability to evade immune surveillance, making it impossible to eliminate. Under the selective pressure of the immune system, the immunogenicity of the remaining tumor cells is further reduced by the production of immunosuppressive factors such as IL-10, TGF-β, PD-L1, and so on, resulting in immune escape.

Despite its significant association with tumorigenesis and progression, smoking has been relatively overlooked in discussions of tumor immunoediting. Therefore, it is imperative to explore the effects of smoking on each stage of tumor immunoediting to understand its influence on tumor development better. By elucidating the impact of smoking on tumor immunoediting, we can gain insights into its role in shaping the tumor microenvironment and inform therapeutic strategies aimed at mitigating its adverse effects.

## TUMOR IMMUNE SURVEILLANCE UNDER THE INFLUENCE OF SMOKING

### Tumor immune surveillance

The initial stage of tumor immunoediting is characterized by the elimination phase, during which the immune system primarily engages in immune surveillance. Upon the emergence of tumor cells, antigen-presenting cells (APCs) such as dendritic cells assemble major histocompatibility complex (MHC) molecules with tumor antigens and present them to effector lymphocytes, including T cells and Nature Killer cells^9,10^. Concurrently, cytotoxic T cells can directly identify and target tumor cells by binding their T cell receptor (TCR) to tumor antigens presented alongside MHC class I molecules on the surface of tumor cells^11^. The collaborative efforts of cellular and humoral immunity are pivotal in recognizing cancerous cells and eliminating them before the tumor progresses to a clinically detectable stage. Therefore, in the subsequent discussion, we will provide a comprehensive elucidation of the roles played by various immune cells in tumor immune surveillance.

*The immune surveillance process is mainly based on cellular immune responses*

CD8+ T cells, also known as cytotoxic T lymphocytes (CTLs), are pivotal effectors in the tumor immune response. They employ two main mechanisms to eliminate tumor cells directly^12^. Firstly, upon contact with target cells, CTLs trigger degranulation, releasing perforin, which forms membrane channels in target cells, facilitating the entry of effector molecules such as granzymes, TNF, and secretory ATP, ultimately leading to target cell death. Secondly, CTLs express Fas Ligand (FasL) upon activation, which binds to Fas receptors on target cells, initiating a signaling cascade that activates DNA degradation enzymes and induces apoptosis. Additionally, CD8+ CTLs can secrete cytokines such as TNF, which indirectly contribute to tumor cell death^13^.

CD4+ T helper (Th) cells play a crucial auxiliary role in tumor immunity. Upon receiving dual signals from APC-presented MHC II complexes and costimulatory molecules, CD4+ Th cells undergo clonal proliferation and release cytokines such as IL-2, IFN-γ, and TNF. These cytokines aid in activating and enhancing the function of CTLs, natural killer (NK) cells, macrophages, and dendritic cells^12^. Furthermore, the discovery of tumor-specific CD4+ T cells, which recognize MHC II-restricted tumor antigens such as tyrosinase, CDC27, gp100, MAGE-3, Melan-A/MART-1, Eph receptor, and NY-ESO-1, underscores their importance in tumor immunity^14-21^. Recent studies have also revealed the cytotoxic capabilities of CD4+ T cells and their role in antigen recognition within the tumor immune microenvironment ^22^.

The γδT cells, characterized by the expression of γ and δ chains and predominantly lacking CD4 and CD8 markers, exhibit non-MHC-restricted cytotoxicity against tumor cells^23^. Although they constitute only a small fraction of peripheral blood lymphocytes, γδT cells are enriched in mucosal tissues and solid tumor-infiltrating lymphocytes (TIL)^24^. Their anti-tumor effects are mediated through mechanisms similar to NK and CTL cells, including the perforin/ granzyme and Fas/FasL pathways. Moreover, γδT cells express NK cell inhibitory receptors, enabling them to regulate their killing activity against tumor cells.

Activated natural killer T (NKT) cells, a specialized subset of T cells expressing T cell-specific markers along with NK cell-specific receptors such as NK1.1 in mice and CD56 in humans, possess potent anti-tumor activity^25^. Upon specific recognition of CD1d molecules, NKT cells rapidly secrete a large number of cytokines in a short period without foreign antigens and participate in anti-tumor immunomodulation^26^. Certain subsets of NKT cells have been shown to play a crucial role in IL-12-mediated anti-tumor immune responses, exerting cytotoxic effects against tumor cells even in the absence of sensitization^25^. NK cells directly eliminate tumor cells through MHC-unrestricted cytotoxicity. Comprising a subset of lymphocytes, NK cells become activated in response to cytokines such as IL-2 and IFN-γ^27^. Their activation is regulated by a balance between killer-cell activating receptors (KARs) and killer-cell inhibitory receptors (KIRs). KARs predominate in the absence or downregulation of MHC class I molecules on target cells^28^. NK cells also exert antibody-dependent cell-mediated cytotoxicity (ADCC) through interaction with tumor-bound antibodies, leading to target cell destruction or apoptosis^27^.

Macrophages contribute to the anti-tumor immune response through multiple pathways^29^. They present antigens to recruit and activate lymphocytes and produce cytotoxic molecules such as hydrogen peroxide, oxygen ions, nitric oxide, and TNF to induce target cell death and engage in ADCC. However, tumor-associated macrophages (TAMs) within the tumor microenvironment exhibit diverse polarizations influenced by the immune milieu, with some subtypes promoting tumor growth^30^: TAMs can be classified into M1 and M2 types based on their cytokine expression profiles. M1-type TAMs exhibit a pro-inflammatory phenotype characterized by high expression of IL-12, IL-23, and inflammatory cytokines, while M2-type TAMs display an anti-inflammatory phenotype with high IL-10 expression and low IL-12 and IL-23 expression. Consequently, M1-type TAMs possess potent anti-tumor activity, whereas M2-type TAMs regulate inflammation and promote angiogenesis.

*Humoral immunity plays a dual role in anti-tumor immunity*

The ADCC pathway and complement-dependent cytotoxic action (CDCs) can eliminate metastatic tumor cells to some extent^31^. Some antibodies have a blocking effect on tumor cells’ surface antigens, such as tumor antigen p185. After binding to its antibody, tumor growth is significantly inhibited. Still, there is also a part of the antigen-antibody binding that reduces the immune system’s ability to recognize tumor antigens, increasing the possibility of immune escape^32^.

*Cytokines make all aspects of tumor immune surveillance interconnected and jointly exert antitumor effects*

IFN-γ plays an important role in anti-tumor immunity, and CD4+ T cells are the most important source of IFN-γ^14^. IFN-γ can promote the secretion of tumor cells chemokines CXCL9 and CXCL10, recruiting CD8+ T cells. On the other hand, IFN-γ can bind to CD8+ T cell surface receptors, promoting their activation and proliferation. While exerting anti-tumor effects, IFN-γ also promotes the immune system to select tumor cells: IFN-γ can promote tumor cell PD-L1 production, thereby inhibiting T cell function^33^. In addition, Cytokines like IL-2, IL-12, IL-23, and TNF-α can be produced by tumor-associated macrophages, dendritic cells, or CD4+ T cells, all of which are paired with CTL, NK cells, Th17 cells proliferation^34^. On the contrary, cytokines such as IL-10 and TGF-β derived from tumor cells and Treg cells inhibit the anti-tumor immune effect^34^.

### Effects of smoking on immune surveillance

**Figure 1:**
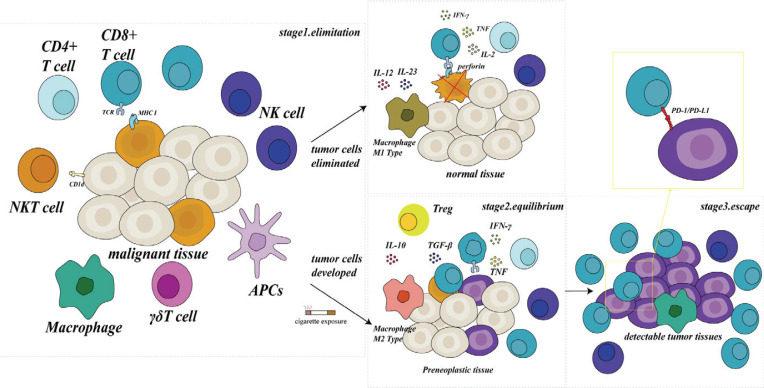
Overview of tumor immunoediting and cigarette exposure. The relationship between the immune system and tumors can be divided into three stages, in early tumor development, the main effect on tumor cells was elimination, due to immune reaction by antigen-antibody recognition mediated and individual immune environment change, some tumors with innate or acquired low immunogenicity evade the surveillance of the immune system to a certain extent, thus entering the equilibrium stage and escape stage. The act of smoking enhances the recruitment of immune cells during the early stage, while also inducing premature tolerance towards cellular immunity. Additionally, smoking creates a more conducive immune microenvironment for tumor growth

One crucial aspect that must be addressed is smoking, before the onset of tumors, serves as a significant inducer of inflammation^35,36^. Comparing smokers and non-smoking individuals in non-tumor populations indicates a higher proportion of immune cell infiltration in samples from smokers^37^. Additionally, non-tumor populations of smokers tend to exhibit a chronic inflammatory environment in the airways, indicative of an activated state in anti-infectious and anti-tumor immune responses^38^. However, excessive activation implies increased cell proportions like Treg cells and an early response in immune suppression mechanisms such as PD-L1^39,40^. This, in turn, momentarily heightens the efficacy of PD-L1 antibodies and other immune checkpoint inhibitors in treatment. These findings align with the views of some studies regarding the enhanced benefits of immunotherapy in lung cancer patients who smoke. Yet, it is noteworthy that tobacco exposure shifts its role from the early phase of immune activation to immune suppression, as the tumor immune microenvironment takes shape. Simultaneously, the early immune hyperactivation triggers the depletion of anti-tumor immune cells. Therefore, from the perspective of tumor immune surveillance, the ultimate effect of smoking leads to a reduction in the quantity of immune cells and inhibition of the anti-tumor effect (Figure 1). For this reason, we have also outlined some of the impacts of smoking on tumor immune surveillance.

*Tobacco exposure can directly impair CD8+ T cells and exacerbate CD8+ T depletion*

Researchers analyzed the results of CD8+ T cells RNA sequencing in smokers and non-smokers. Smokers were found to have decreased expression of granzymes B in CD8+ T cells, and further analysis found that smoking reduces expression of CD8+ T cells IL-2 receptors by miR-629-5p epigenetic modification, thereby attenuating CD8+ activation of T cells^41^. In addition, in the period before the tumor occurred, smoking caused an inflammatory immune response in the body, which can cause an increase in the level of CD8+ T cells; some studies report that this inflammatory immune response causes CD8+ T cell depletion^42^. A study on CD8+ T cells telomerase found that telomerase activity significantly decreases in smokers with COPD, suggesting that CD8+ T cells are senescent in chronic inflammation caused by long-term tobacco exposure^43^, which supports the idea that smoking causes CD8+ T cell depletion.

*Tobacco exposure decreases CD4+ T cell levels and induces Th2 subset differentiation, leading to immune microenvironments that facilitate tumor growth*

CD4+ Th cells, which play a part in the recognition of tumor MHC II molecules, have been reported to be decreased over a long-time tobacco exposure in both alveolar lavage fluid and peripheral blood^44^. What is more, tobacco exposure also broke the balance of the Th1 and Th2 subsets and induced Th2 cell differentiation^45^. With the number of Th1 cells decreased, cytokines like IFN-γ decreased and obstructed recruitment and activation of CTLs in cancer^46^. This result eventually led to a chronic inflammation microenvironment more conducive to tumor growth.

*Tobacco exposure can active regulatory T cells (Treg) that inhibit CD8+ T cells*

Regulatory T cells (Treg) play a vital role in maintaining immune homeostasis and tolerance through their immunosuppressive capacity^47^. Compared with healthy non-smokers, the percentage of CD4+, and CD25+ Tregs is increased in alveolar lavage fluids of smokers and COPD patients^48^. In addition, epidemiological investigations revealed smoking during pregnancy was significantly associated with an increased tumor incidence in offspring. Further studies found that tobacco exposure during pregnancy significantly increased Treg cells and TGF-β levels in offspring, thus inhibiting CD8+ T cell function, increasing the risk of tumors^49^.

*Tobacco exposure has an inhibitory effect on the ability of DC cells to present antigens*

DC participates in the immune response by processing and presenting antigens of the pathogen or tumor^50^. However, some studies suggested that smoking impairs the function of dendritic cells: tobacco exposure reduces the antigen-presenting capacity of DC in asthmatic mice^50^, and carbon monoxide exposure reduces DC recruitment of CD8+ T cells^50^.

*Tobacco exposure inhibits NK cell activity by lowering peripheral blood IL-15 levels*

The peripheral blood mononuclear cell system produces IL-15 and has an activating effect on NK cells. Studies have shown that tobacco exposure reduces peripheral blood IL-15 levels, thereby affecting NK cell activation^51^.

*Tobacco exposure affects macrophage antigen presentation and induces tumor-associated macrophage (TAM) differentiation into type M2*

Early studies found that smoking had a reversive effect on M1 type TAM, leading to TAM’s preference for M2 type^30^. The M2 tumor microenvironment is characterized by the co-expression of CD68 and CD163, and high levels of CD163 expression in macrophages are associated with mortality of malignant tumors, including lung adenocarcinoma^52^. Further studies revealed that the expression of CD 163 is correlated with high levels of macrophage colony-stimulating factor (CSF1), which is a ligand of CSF1R, a tyrosine kinase receptor expressed in tumorassociated macrophages (TAM) and can mediate tumorigenesis through keeping TAM in an M2 type^52^. Inamura et al.^53^ found that CSF1R expression in non-smokers was more strongly associated with mortality.

*Cigarette extract inhibits the secretion of anti-tumor cytokines*

*In vitro* experiments have shown that cigarette extract inhibits cytokines secreted by peripheral blood macrophages, and the inhibitory effect on IL-1β, IL-2, IFN-γ, and TNF-α is more than 90%^51,54,55^.

In conclusion, the relationship between smoking and immune surveillance is characterized by chronic inflammation induced by smoking, leading to a sustained state of immune activation. This chronic inflammation results in elevated levels of immune regulatory factors such as TGF-β and PD-L1, contributing to T cell exhaustion and providing a rationale for the potential benefits of immunotherapy in some smoking patients. However, smoking directly impairs the immune system, compromising the function of cellular immunity and creating an immune environment conducive to tumor growth. The prematurely activated immune system also reinforces selective pressure on tumor cells, further shaping the tumor microenvironment.

## HOW SMOKING LEADS TO TUMOR IMMUNE ESCAPE THROUGH SHAPING TUMOR CELLS

### Mechanism of tumor immune escape

Variations in immunogenicity exist among tumor cells. Those with robust immunogenicity can trigger a potent anti-tumor immune response, swiftly eliminated by the body. Conversely, tumor cells with comparatively weaker immunogenicity can evade immune surveillance and proliferate selectively, a phenomenon termed immunoselection^7^. Here are some alterations in tumor cell phenotype and mechanisms linked with tumor immune evasion.

*Loss of CTLs function*

CTLs, the most crucial immune cells in anti-tumor responses, play a pivotal role. Any disruption in their functioning at even the minutest level can result in tumor immune evasion^56^: disturbances in CTL recognition, downregulation of tumor cell surface molecules such as MHC or co-stimulatory molecules expression, leading to escape from recognition of tumor cell surface antigens and subsequent evasion from host immune system attack.

*Fas/FasL pathway disorder*

There are two main obstacles to this pathway^57^: 1) tumor cell Fas transcriptional downregulation or deletion, Fas/FasL pathway-mediated apoptosis of tumor cells weakens or disappears; 2) T cells express Fas molecules on the surface, while some tumor cells express FasL, attacking T cells in reverse, causing T cell apoptosis, further increasing the risk of immune escape.

*Changes in tumor antigens – a type of antigen modulation*

Tumor antigens were once divided into tumor-specific antigen (TSA) and tumor-associated antigen (TAA), which have already had a wide application in clinical testing and therapy. With the appearance of next-generation sequencing and the development of immunoediting theory, the concept of tumor neo-antigen arose. Tumor neo-antigen, a class of antigens unique to tumor cells, were mainly derived from point mutations in the tumor cell genome (about 95%), gene insertion or knockout, and frameshift and structural mutations (about 5%)^58^. The neo-antigen can be combined with MHC I and MHCII^11,21,59^, thus recognized by T cells and promoting further anti-tumor immune response. Nonetheless, not all neoantigens had immunogenicity^58^. In the force of immunoselection, some tumor cells with neo-antigens that the immune system cannot recognize were rested, and eventually, most existing neo-antigens were unrecognizable. The progress of these changes in tumor antigens was also a type of tumor antigen modulation.

*Tumor cells suppress anti-tumor immunity by synthesizing immunosuppressors*

Tumor cells can secrete various immunosuppressive factors, including IL-10, TGF-β, and GM-CSF 34 cytokines. TGF-β is the most powerful inhibitory cytokine, which can antagonize the IL-2 activation effect of NK cells and CTL. GM-CSF promotes the proliferation of tumor-associated macrophages. Tumor cells can also produce small molecules such as PGE2 and ROS, down-regulating HLA-DR inhibiting cellular immunity. In addition, IL-10, TGF-β, and PGE2 can inhibit the maturation of dendritic cells and the expression of MHC II and B7 co-stimulating molecules on their surface, thereby influencing the recognition of the tumor. The production of the immunomodulatory ligand PD-L1 in tumor cells is another important cause of immune escape. The production of PD-L1 is a negative feedback effect, which essentially inhibits the production of an excessively strong immune response^60^. NK cells, CTL, or Th1 cells release IFN-γ, which activates the JAK-STAT-IRF1 signal pathways that increase the expression of PD-L1 in tumors. After PD-L1 binding T cell surface PD-1, the two major signaling pathways PI3K-Akt and Ras-MEK-ERK are activated and inhibit the CTL activation, proliferation, and even mediating T cell death^61^.

### Smoking affects tumor cells and thus changes tumor immunoediting outcomes

**Figure 2:**
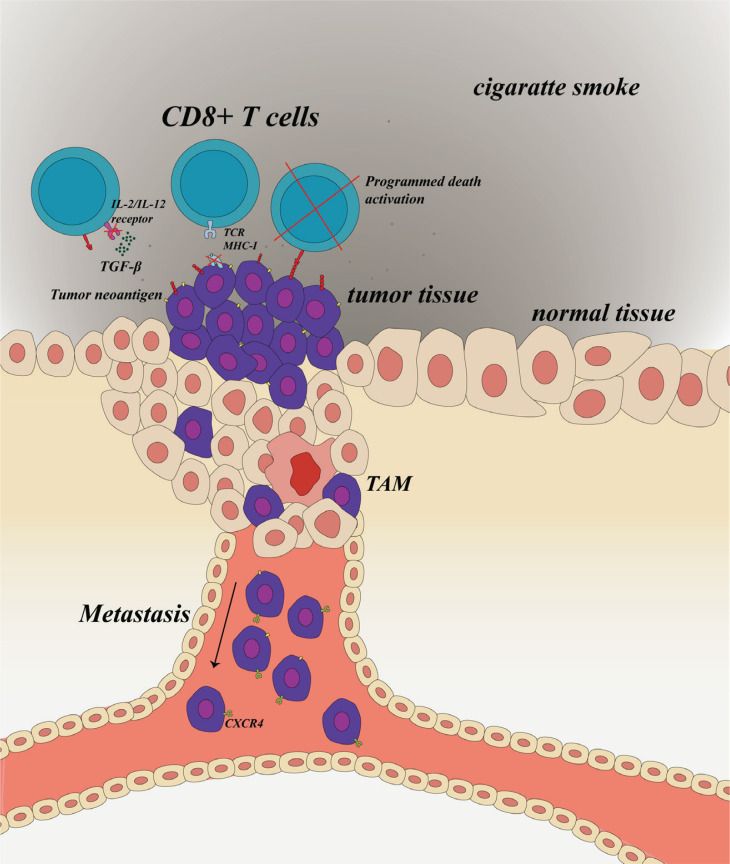
Smoking affects tumor immunity by shaping tumor cells. Smoking exposure inhibits the MHC I molecules and enhances the expression of PD-L1 and TGF-β in the immunity microenvironment. With the increased TGFβ signaling, CXCR4 is upregulated which may cause malignant cells to migrate

Tobacco exposure significantly influences the genetic instability of tumor cells (Figure 2). Prolonged exposure to tobacco leads to the release of substantial quantities of oxygen-free radicals in the body, directly inducing DNA fragmentation and causing damage to purines, pyrimidines, and deoxyribose through reactive oxygen species (ROS)^62^. Additionally, regulated signaling pathways such as NF-κB may be activated due to tobacco exposure^63^. Furthermore, tobacco exposure mediates the action of APOBEC cytidine deaminase, resulting in DNA damage, errorprone replication, and indirect activation of DNA editing processes^64^. These alterations inhibit the expression of tumor suppressor genes while activating proto-oncogenes, thereby modifying the proliferative state of tumors. Consequently, this process induces tumor antigen modulation and increases the burden of anti-tumor immunity^34,65,66^.

*Smoking can inhibit the recognition of MHC I molecules*

Smoking affects the function of MHC I molecules on the surface of tumor cells through various pathways: tobacco exposure activates the ROS/NF-KB signaling pathway to improve the expression of PCSK9^67^. Studies have shown that the expression of the PCSK9 gene activates lysosomes and promotes the degradation of MHC I molecules on the cell surface, thereby reducing tumor immune cell infiltration and increasing the possibility of immune escape^68^. In addition, smoking has a negative regulatory effect on the function of immunoproteasomes (LMP), which play an important role in the assembly and antigen presentation of MHC I molecules^63,69^. This triggers MHC I dysfunction and affects the immune system’s recognition and clearance of tumors.

*Smoking can induce neoantigen expression in tumor cells, thus influencing anti-tumor immunity*

Researchers have revealed a positive correlation between tumor mutant burden (TMB) and neoantige^65,70^, and the relationship between smoking and TMB is certain^64^. Wang et al.^65^ found TMB was positively related to smoking in lung adenocarcinoma, and it may be associated with RYR2 mutant caused by smoking. In head and neck squamous cell carcinoma (HNSC) and lung squamous cell carcinoma (LUSC), smoking also increased TMB^71^. Furthermore, by comparing smoking and non-smoking NSCLC patients, a clinical study found that the increase of TMB is dose-dependent on smoking^71^. The misreplication of DNA damage and indirect activation of DNA editing by APOBEC cytidine deaminases^62^ explained the potential mechanism that smoking increased TMB. In a word, both basic research and clinical research have shown that smoking enhanced the TMB, which has a positive correlation with tumor antigen expression.

With a higher level of tumor neoantigens, more CTL will infiltrate the microenvironment, enhancing IFN-γ level, thus programmed death ligand-1 (PD-L1) molecules^5,60,61,72^ and cytotoxic T lymphocyte-associated antigen-4 (CTLA-4) increasing^73^. Meanwhile, monoclonal antibodies against PD-L1, such as pembrolizumab, had a better curative effect on the part of smoking cancer patients^5,58,74^. However, this progress also accelerates antigen modulation of tumor cells.

*Smoking can directly increase the expression of PD-L1 in tumor cells and increase immune escape*

Many studies have found that squamous cell lung carcinoma patients with a history of smoking have a higher level of PD-L1 expression^63,75-77^. Besides the reason for CTL infiltration, long-term smoking causes chronic inflammation and activates inflammatory pathways, thus increasing the expression of PD-L1^63^. Wang et al.^75^ found a cell membrane receptor binding to benzopyrene, named aromatic hydrocarbon receptor (AhR), which inhibits tumor development after blocking and is associated with the expression of PD-L1. Previous studies have also found that the binding of tumor cells AhR to ligands can cause activation of the PD-L1 pathway^78^. This series of studies suggests that benzopyrene and tumor cell AhR in tobacco cause elevated PD-L1 levels, which may be another potential mechanism for smoking to increase tumor immune escape.

*Smoking enhances the secretion of TGF-β*

Transforming growth factor β (TGF-β) is the main inhibitory cytokine produced by tumor cells^79^. It significantly inhibits the IL-12 and IL-2, obstructing T cell proliferation^80^. Though there is no evidence suggesting tobacco element exposure directly stimulates malignant cells, the stimulative effect of smoking on TGF-β levels in the immune microenvironment is certain^81,82^. With the increased TGF-β signaling, C-X-C motif chemokine receptor 4 (CXCR4) is upregulated, which may cause malignant cells to migrate^83^.

## DISCUSSION

Smoking is an important risk factor for tumorigenesis^3^. However, studies have shown that some tumor patients with a history of smoking have a better anti-PD-L1 treatment effect^5^, which calls into question the status of smoking as a tumor risk factor. The discovery of tumor cell immunomodulatory ligand PD-L1 is a major achievement in tumor immunity, and the advent of its blocking drugs has also made tumor therapy enter a new stage^34^. It has greatly improved the prognosis of patients with PD-L1 high-expression tumors^5^. Although smoking patients have a better prognosis in anti-PD-L1 treatment, it is not rigorous to conclude that smoking is beneficial to tumor patients.

Our literature review provides an explanation of this phenomenon from the perspective of tumor immunoediting, further reaffirming the promotional role of smoking in cancer.

### Limitations

Our study has some limitations. Firstly, being a literature review, we lack first-hand experimental or clinical data to support our viewpoints. Secondly, in the context of tumor immune editing theory, the phase of tumor immune surveillance does not clinically manifest as tumor formation, making research during this stage challenging. We have, therefore, selected some studies on long-term smoking exposure or chronic obstructive pulmonary disease as supportive evidence, which may have certain limitations.

## CONCLUSION

From the perspective of tumor and immunity, this review introduces various stages of tumor immune editing and the impact of smoking: the process of tumor immune editing can be divided into the surveillance period (elimination phase), equilibrium period, and escape period^7^. In each period, the role of the immune system on tumors is not the same. The surveillance period is mainly to eliminate the role of tumor cells, and from the equilibrium period to the escape period, the immune system plays a screening role on tumor cells, and a group of ‘surviving’ tumor cells eventually develop into clinically visible tumors.

In general, tobacco exposure provides a breeding ground for tumor growth. Although, at first, the inflammatory response caused by smoking enhances the responsiveness of the immune system to the outside world. Eventually, smoking participates in the exhaustion of anti-tumor immunity, it depletes the function and number of anti-tumor immune effector cells in immune surveillance. On the other hand, tobacco exposure increased tumor mutation load, making tumor cells become more heterogeneous, some changes activating the proliferation of somatic cells, and some further shaped them to lower immunogenicity, including tumor neoantigen modulation, inhibiting tumor cell MHC molecules, as well as promoting the expression of immunosuppressive factors. Therefore, no matter in which stage of tumor immunoediting, the impact of smoking is to promote the occurrence and development of tumors.

## Data Availability

The data supporting this research are available from the authors on reasonable request.
